# Medical and Physical Intelligence

**Published:** 1825-11

**Authors:** 


### MEDICAL AND PHYSICAL INTELLIGENCE.

### ANATOMY.

1. *Origin of the Spinal Nerves.*—M. AMUSAT announces the following facts as certainly established:—1. That the ganglion which is situated not far from the origin of each of these nerves belongs exclusively to its posterior root, and that the threads of the anterior roots are only coiled round it. 2. That this ganglion is not formed of a homogeneous tissue. 3. That the nervous filaments are not interrupted at this spot; but that they are perfectly continuous, only separated the one from the other, and reinforced by the grey substance; and that they form a larger bundle in going out than on entering the ganglion. 4. That the filaments of each root agree in such a manner in their ulterior divisions, that the small nerve contains a thread of each: one destined for sensation, the other for motion.

M. Amusat also announces that the nerves of the sacral plexus, in reuniting to form the sciatic nerve, cross each other in pairs.—(*Revue Medicale*, Août.)

---

### PHYSIOLOGY.

2. *Chyle in the Veins of the Jejunum.*—Dr. MEYER, professor at the University of Bonn, relates the following case:—A robust man, aged about seventy-nine years of age, died suddenly in consequence of asthma; and his body was taken to the dissecting-room. This man had been healthy from his youth: he had never suffered from any severe illness; only, in the course of long military service, he had experienced some cutaneous affections, particularly the itch. He was a great brandy drinker. About six months before his death, he had complained of his chest, of palpitations of the heart, of jaûndice, with symptoms of dropsy; but, by proper remedies, he was so much recovered as to return to his usual habits. At the end of three months, his dropsical symptoms returned, with a purulent expectoration, and œdematous feet. He again became a little better; but shortly afterwards he suddenly felt violent darting pains in the chest, with violent cough, and other symptoms of inflammation of the lungs. He was bled, and the blood exhibited the buffy coat. He felt relieved the following day; but, a few hours after having taken some soup, he died suddenly.

The examination of the body showed the following particulars:—A great quantity of serum in the chest, as well as in the pericardium; the lungs adhering to the parietes of the thorax, and full of tubercles, with small cysts containing pus. There was no remarkable alteration in the viscera of the abdomen. The intestines were then taken out, and preserved for the purpose of demonstration. When the different portions of them were submitted to especial examinations, on the small intestines vessels of a greyish white were perceived, a little inclining to yellow in some parts, and equally visible on the internal surface of the canal as on the outside. At first they were taken for lymphatics; but a closer examination proved them to be veins. The lower third of the duodenum, the entire jejunum, and a portion of the ileum, were spread over with similar venous ramifications, containing a liquor analogous to cheese: nevertheless, this liquor only existed in the minute ramifications upon the surface of the intestines; but, at the spot where the mesentery is inserted, the venous trunks to which these smaller vessels conducted, contained only common blood. Upon many points might be seen, with the naked eye, the little roots of the white veins advance to the edge of the valves of Kerking; and it appeared to the Doctor that, pressing lightly, he drove the white liquor contained in the venous branches to the surface of the mucous membrane. The small intestines were full of chyme. No lymphatic filled with chyle was to be found. Unfortunately, the other abdominal organs were not in a state to permit of examination.

It is not to be doubted, continues Dr. Meyer, that the greyish liquor, which was contained in the smallest branches of the veins, was truly chyle, the colour and consistence of which are in general relative to the quantity of chyme. But it may be asked, how did it happen that the lymphatic vessels contained no chyle, since the patient appeared to have died in the moment of chylification. Circumstances do not permit him to resolve this question; but he conjectures that the venous system may have outlived the lymphatic. Perhaps, the right side of the heart had outlived the left: in this case there would have been a prolonged diastole and systole of the right ventricle, which might have determined the absorption of the chyle by the smaller venous branches of the small intestine. The appearance of the heart rather corroborated this hypothesis. (*Journal Comp.* Août.)

---

MORBID ANATOMY.

3. *Aneurismal Diathesis.*—M. Chomel presented to the Royal Academy of Medicine a heart, with its aorta, upon which that alteration of tissue constituting the principle of aneurism was found extended over a considerable surface. The heart was rather larger than natural; the parietes of the right ventricle thicker than those of the left. The aorta was uneven throughout its whole extent; and these inequalities were caused by white patches, semi-cartilaginous, which formed the greater part of its surface. From its origin to the distance of three inches and a half, it presented three hollows, circumscribed by a marked projection; one on the right side, a second on the left, and a third above the former. These depressions, from six lines to an inch broad, and from two to eight lines in depth, had only cellular tissue for their parietes, or the internal tunic of the artery thickened, and preserving no distinct structure. Even this tissue had suffered the same alteration in a portion of its circumference. (*Revue Medicale*, Août.)

4. *Softening of the Stomach.*—M. Ferries presented to the Royal Academy of Medicine two cases of softening of the stomach. In one, the alteration was not announced during life by any remarkable symptom, which renders it more doubtful whether it always be a consequence of inflammation: the patient was merely affected with scurvy. Could that disease have been the cause of the softening? (*Ibid.*)

5. *Rupture of the Heart.*—The same gentleman also presented a heart, having on its surface several scorbutic spots, and a rupture, one inch in length, in the left ventricle, near its apex. At the spot where the rupture was found, the substance of the heart appeared changed; and it was evident that the rupture had taken place from within outwards. The patient was the same whose stomach has just been described as softened. A little before his death he had received a fall, and had fractured three ribs on each side, and ever after experienced a feeling of choking, with a sensation of heat at the præcordia. (*Ibid.*)

6. *Alteration in the Bones of the Head.*—A report was made by MM. EMERY, RIBES, and MURAT, upon a diseased cranium, presented by M. DUVERGIE. This cranium belonged to a veteran, who had died of disease of the heart. There was a considerable thickening of all the anterior part of the frontal bone, of the ethmoid, and part of the sphenoid bone. The projections which the internal plates of the frontal sinuses and the crista galli made, had displaced the optic nerve on one side; and, nevertheless, no lesion of vision had occurred during life. *(Ibid.)*

### PATHOLOGY.

7. *Non-Contagious Nature of Yellow Fever.*—Dr. COSTA read, before the Royal Institute of France, a Memoir on this subject. The facts and observations related in this work have been collected from the sources of the disease; Dr. Costa having been physician to a lazaretto. After describing the state of affairs at Barcelona, and the precautionary measures adopted on the frontiers, which he considers as very inefficient, had contagion really existed, the author does not hesitate to affirm that the disease is not contagious. Nay, so persuaded is he of this fact, that, together with MM. LASSIS and LASSERRE, he proposes to make experiments on the subject. He requests that clothes which have been worn by persons who have died of the disease, may be sent to him from the Havannah, or wherever the disease is found to exist: these clothes, hermetically sealed during the passage, shall be worn by these gentlemen, without making use of any disinfecting medium. *(Revue Medicale,* Août.)

### THERAPEUTICS.

8. *On the Use of Injections containing Ammonia in Cases of Amenorrhœa.*—GRAFE and WALTHER's Journal contains a paper upon the injection of liq. ammoniæ into the vagina, in cases of suppressed menstruation. In fourteen cases, it succeeded eight times. The menses returned; but the affection upon which the suppression had originally depended in most of them remained unrelieved. From fifteen to twenty-five drops of the liq. ammon. caustici, mixed with two tea-spoonsful of milk, warmed to 28° of Reaumur, was employed. Where it was possible, the fluid was injected into the os uteri. *(Journal für Chirurgie, &c.* band 7, keft 2, 229.)

### SURGERY.

9. *Successful Case of High Operation for the Stone;* by A. COPLAND HUTCHISON, Esq.—The patient being placed on a mattress upon a table of ordinary height, with a pillow under his head; the pelvis raised considerably higher than his shoulders, with the view of removing the peritoneum as far from the parts to be cut as possible; while his feet rested upon chairs; standing on the right side of the patient, I made an incision, nearly four inches in length, with a scalpel, in the line of the linea alba through the integuments downwards over the front of the pubes; which latter step I found of great advantage, both during the operation and in the subsequent treatment of the case. The incision was carried on down towards the bladder, between the pyramidales muscles, and through the linea alba; the latter being first punctured, and afterwards cut *transversely upon the symphysis pubis,* dividing some of the fibres of the pyramidal muscles on each side as we proceeded, so as to give a more free passage for the extraction of the stone. I then, with a director, detached this tense membrane (the linea alba) from its subjacent adhesions, and divided it upwards with a probe-pointed bistoury, introduced into the groove of the director, until I could readily insert the fore-finger of my left hand, which is, on all occasions, the best director; and which was most conveniently done by standing between the legs of the patient. When about two inches and a half of the linea alba were thus divided, I thought we should have had sufficient space for cutting into the bladder, and extracting the stone; but, as the wound appeared deep and narrow, and there was a large calculus to extract, I cautiously extended the incision half an inch more, in the manner already described.

The bladder, very much contracted, was now clearly seen covered by fat, and was raised through the fat, upwards and forwards, to the external wound of the integuments, upon the point of a silver staff, with a groove in its concave part, extending from close to its point downwards about two inches, and which I preferred to the *sond* used by the French surgeons, that instrument appearing to me both unnecessary and unscientific. The handle of the silver staff being held in a depressed position by my friend Mr. Brown, the anterior part of the fundus of the bladder was pierced from without, by a straight sharp-pointed bistoury, passing its point into the groove of the staff, and carrying the incision downwards and forwards to the pubes, until there was room for the fore-finger, which was introduced for the purpose of ascertaining the size of the calculus, that the opening in the bladder might be enlarged accordingly. Having satisfied myself on this head, I hooked up the fundus of the bladder with my finger, and enlarged the incision towards its cervix, sufficiently to admit of the free extraction of the stone. Some little difficulty here arose in disengaging the stone from its situation, notwithstanding the great assistance obtained by the introduction of the fore-finger of my right hand into the rectum, while the fore-finger of my left was in the bladder, to cant or turn the stone, so as to extract it by its smaller axis: and, indeed, this was not effected until the single blade of a pair of small stone-forceps was introduced. The stone being thus turned, it was easily removed with the finger and thumb. In point of fact, so firmly did the bladder grasp the calculus, that the idea was conveyed to our mind of its actual adhesion to the coats of that viscus.

One small cuticular artery was divided at the first incision, and secured at the time, to prevent the future steps of the operation being obscured by the bleeding, and the subsequent issue of blood into the cavity of the pelvis.

Two slips of linen, dipped in oil, were introduced through the external wound on each side of, but not into, the bladder. A gum-elastic catheter was passed into the bladder, so as just to enter its cavity, and no further, and was secured in this situation by tapes attached to the instrument, and to an elastic band, or retainer; which in this case was made of flannel, lined with calico. The sides of the wound were brought together by slips of sticking-plaster, and the parts supported by a flannel roller, passed three or four times round the pelvis and the lower part of the abdomen. The patient, by means of pillows, was placed on an inclined plane; a vessel was secured to the end of the catheter to receive the urine; and he had an anodyne draught administered to him.

I am fully aware that there are those who may imagine the trifling difficulty we experienced in extracting the stone, arose from the depth, narrowness, and consequently from the supposed tightness, as it were, of the wound, through the parietes of the abdomen: but I can assure them that such was not the fact, for I enjoyed the most perfect freedom in that respect. The circumstance, in my mind, arose rather from the position of the stone, its size, and the strong contraction of the bladder upon it: for it will be borne in recollection that the bladder, in this case, had never been distended beyond the size of the calculus, and that which two ounces of urine would occasion. What the bladder wanted in capacity, it possessed in the increased thickness of its parietes, for it was fully a quarter of an inch thick at the part divided; so that its muscular power must, therefore, have been considerable. I cannot avoid, in this place, repeating the great advantage to be derived from dislodging the stone in such cases, by the introduction of the finger into the rectum, and which appears to me a great improvement in this mode of operating.* (*Letter to* Sir E. HOME, Bart.—*Journal of Science*, October.

---

PHARMACY.

10. *Preparation of Lactucarium.*—M. ROMAN, after commenting upon the method followed by M. FRANCOIS in the preparation of this medicine, says that he prepares it in the following manner :—He took thirty pounds of lettuce in flower, and then deprived all the stems of their flowers and tops. After cutting them in pieces, they were bruised in a marble mortar, and afterwards placed to macerate in six littres of water; they were then strained, and left to settle; and, after this, boiled for some minutes, in order to separate the greenish fecula formed during ebullition; the liquor was then filtered, and evaporated to a proper consistence. From the above quantity of lettuce were obtained six ounces of a reddish-brown extract, of a resinous appearance, attracting humidity from the atmosphere, and of a taste analogous to that of opium. Its properties were the same as those of the thridace of M. Francois. Administered in the same doses, it produced the same results. (*Revue Medicale*, Août.)

---

* The patient did well, recovering very speedily.

11. *Sulphate of Quinine.*—M. CAVENTON made a report to the Royal Academy of Medicine, relative to a Memoir of M. GUERETTE, of Toulouse, respecting the sulphate of quinine, obtained from the residuum of the cinchona bark, left after making the decoction. M. PELLETIER says, that the bark treated with boiling water, although it has lost part of its quinine in the form of quinic acid, retains still a good deal, in the state of a sub-salt; and, in fact, bark that has been treated with water in the ordinary method, affords a very fine sulphate of quinine, which is white even on its first crystallisation. The watery extracts and decoctions of cinchona possess, then, a small quantity of quinine; and it appears that it exists in combination with the quinic acid.

M. Pelletier has also obtained the quinine in a state of crystallisation; and, by dissolving it in alcohol of from 40 to 42°, and placing this in a cold but not a damp situation, he has seen the quinine crystallised in silken tufts, or in elongated prisms.

M. ROBIQUET has also found that, on adding a mineral alkali to a boiling solution of the sulphate of quinine, this separates by the cooling of the liquor in black plates, and forms beautiful ramifications. (*Archives Generales*, Juillet.)

### METEOROLOGICAL JOURNAL,

By Messrs. William Harris and Co. 50, Holborn, London.

*From September 20 to October 19, 1825.*

| | Moon. | Rain gauge | Therm. | | | Barom. | | De Luc's Hyg. | | Wind. | | Atmospheric Variations. | | |
|---|---|---|---|---|---|---|---|---|---|---|---|---|---|---|
| | | | 9 A.M. | Max. | Min. | 9 A.M. | 10P.M. | 9 A.M. | 10P.M. | 9 A.M. | 10P.M. | 9 A.M. | 2 P.M. | 10P.M. |
| Sep. | | | | | | | | | | | | | | |
| 20 | | | 65 | 71 | 62 | 29.65 | 29.67 | 81 | 80 | WSW | SW | Fine | Fine | Fine |
| 21 | | | 66 | 69 | 57 | 29.47 | 29.50 | 85 | 75 | WSW | WSW | Rain | | |
| 22 | | .17 | 62 | 69 | 49 | 29.52 | 29.74 | 75 | 76 | WNW | NW | Cloud. | | |
| 23 | | | 54 | 69 | 59 | 29.81 | 29.92 | 72 | 77 | W | SW | Foggy | | |
| 24 | | | 63 | 70 | 63 | 29.90 | 29.93 | 76 | 87 | WSW | WSW | Fine | | Cloud. |
| 25 | | | 66 | 70 | 62 | 29.93 | 29.89 | 85 | 68 | W | WSW | Cloud. | | |
| 26 | | | 65 | 69 | 50 | 29.83 | 29.78 | 61 | 61 | WSW | WNW | Rain | | |
| 27 | ○ | .53 | 60 | 64 | 48 | 30.01 | 30.14 | 69 | 75 | W | NNE | Fine | | |
| 28 | | | 54 | 64 | 50 | 30.23 | 30.18 | 80 | 69 | NE | ESE | | | |
| 29 | | | 58 | 60 | 46 | 30.14 | 29.98 | 55 | 65 | ESE | ESE | | | |
| 30 | | | 50 | 58 | 55 | 29.85 | 29.78 | 70 | 76 | SSE | SE | | | Rain |
| Oct. | | | | | | | | | | | | | | |
| 1 | | 40. | 56 | 61 | 58 | 29.74 | 29.67 | 89 | 77 | ESE | S | Rain | Fine | Sho'ry |
| 2 | | | 62 | 60 | 55 | 29.60 | 29.60 | 85 | 85 | S | S | Rain | Rain | Rain |
| 3 | | .25 | 59 | 63 | 57 | 29.54 | 29.54 | 89 | 85 | SSE | S | Rain | Rain | Fine |
| 4 | | | 60 | 65 | 55 | 29.70 | 29.89 | 87 | 80 | SW | SSW | Cloud. | Fine | Fine |
| 5 | ☾ | | 60 | 67 | 58 | 29.98 | 30.00 | 84 | 75 | SW | SSW | Fine | | Cloud. |
| 6 | | | 63 | 67 | 55 | 29.90 | 29.74 | 80 | 83 | SW | S | Rain | Fine | Rain |
| 7 | | .62 | 57 | 65 | 47 | 29.45 | 29.88 | 73 | 76 | SW | W | Fine | Fine | Fine |
| 8 | | | 54 | 64 | 51 | 29.90 | 29.74 | 72 | 76 | SW | SSW | Fine | Cloud. | Cloud. |
| 9 | | .38 | 55 | 62 | 58 | 29.85 | 29.90 | 75 | 75 | W | W | Fine | Cloud. | Cloud. |
| 10 | | | 60 | 64 | 57 | 30.10 | 30.13 | 74 | 83 | W | WSW | Cloud. | Cloud. | Cloud. |
| 11 | ● | | 61 | 69 | 58 | 30.22 | 30.18 | 82 | 82 | WSW | SW | Foggy | Fine | Fine |
| 12 | | | 62 | 66 | 50 | 30.03 | 30.06 | 85 | 80 | SW | NW | Foggy | Fine | Foggy |
| 13 | | | 55 | 62 | 50 | 30.02 | 30.02 | 82 | 70 | W | WSW | | Fine | Cloud. |
| 14 | | | 52 | 60 | 46 | 30.10 | 30.08 | 79 | 80 | NW | W | Fog | Fine | Sho'ry |
| 15 | | | 51 | 58 | 44 | 30.29 | 30.35 | 70 | 73 | WNW | NW | Fine | Fine | Fine |
| 16 | | | 54 | 58 | 44 | 30.35 | 30.22 | 72 | 76 | WSW | SW | Fine | Fine | Fine |
| 17 | | | 47 | 58 | 42 | 30.13 | 29.95 | 80 | 74 | WSW | WNW | Foggy | Rain | |
| 18 | ☽ | .67 | 46 | 53 | 47 | 29.99 | 29.51 | 75 | 85 | W | WSW | Fine | | Rain |
| 19 | | | 50 | 63 | 37 | 29.31 | 28.94 | 72 | 78 | WNW | S var. | Cloud. | | |

The quantity of rain fallen in the month of September, was 2 inches and 28.100ths.

